# The Effects of Different Drying Methods on the *In Vitro* Bioaccessibility of Phenolics, Antioxidant Capacity, and Morphology of European Plums (*Prunes domestica* L.)

**DOI:** 10.1021/acsomega.3c08383

**Published:** 2024-03-08

**Authors:** Elif Yener, Oznur Saroglu, Osman Sagdic, Ayse Karadag

**Affiliations:** † Department of Food Engineering, Faculty of Chemical and Metallurgical Engineering, Yildiz Technical University, 34210 Istanbul, Turkey; ‡ Food Institute, TUBITAK Marmara Research Center, Gebze 41470, Turkey

## Abstract

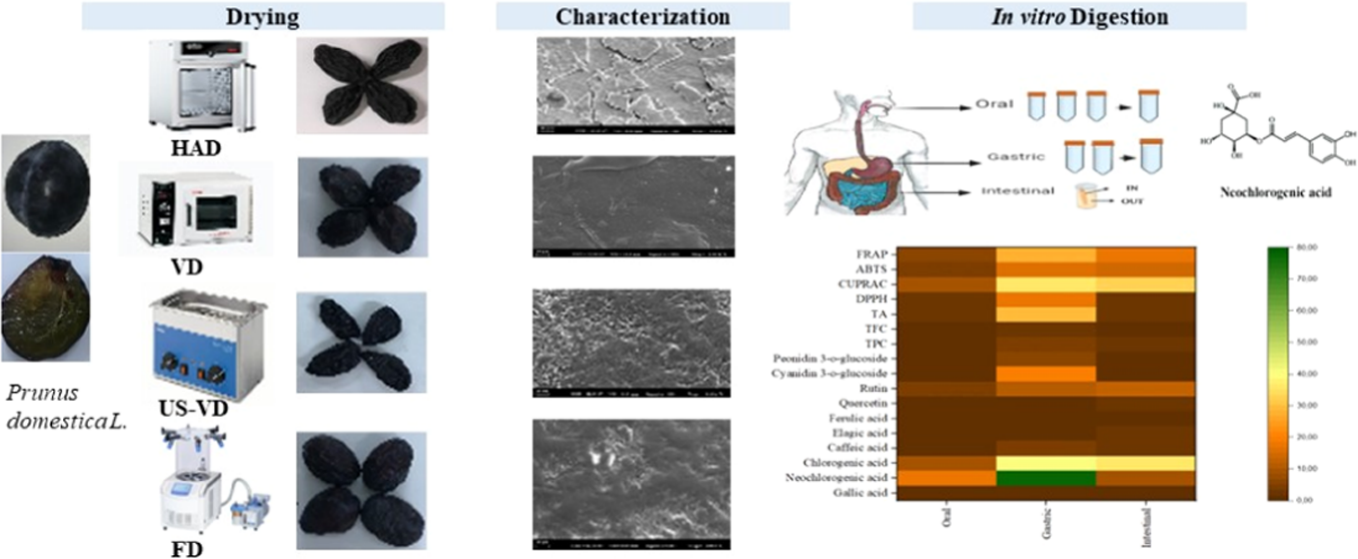

Four different drying methods, hot-air-drying (HAD), vacuum-drying (VD), ultrasound-assisted vacuum-drying (US-VD), and freeze-drying (FD), were used to obtain dried plums (*Prunes domesticaL*.). These prunes were evaluated for their physical properties (such as color, rehydration ratio, and microstructural properties), phenolic compounds, and antioxidant activities before and after being subjected to *in vitro* digestion. TPC (total phenolic content) of plums ranged from 196.84 to 919.58 mg of GAE (gallic acid equivalent)/100 g of dw, and neochlorogenic acid was the most abundant phenolic compound. FD prunes had the highest levels of phenolics, whereas US-VD caused the most significant loss. During *in vitro* digestion, the phenolics were present at higher levels at the gastric medium but failed to maintain their stability at the small intestinal stage. Among the samples, FD along with HAD prunes exhibited a higher bioaccessibility index for most of the phenolic compounds. The ratios of TPC, TFC (total flavonoid content), and individual phenolics determined in the digested residues to the initial values of the undigested samples ranged from 0.23 to 31.03%. It could be concluded that the majority of the phenolics were extracted during digestion. Our findings showed that the different drying methods would alter the microstructure, which would affect the extractability and release of phenolics in the simulated digestion model.

## 1 Introduction

The European plum (*Prunes domestica*) contributes 20% of the global plum production.^1^ Although plums are rich in nutraceuticals with bioactive properties, their high moisture content (around 80%) makes them very susceptible to chemical, physical, and microbiological damage, which restrains their market availability.^2^ Drying has been one of the major preservation methods for extending the shelf life of fresh fruits. Prunes, the dried form of plums, have been reported to contain health-promoting phytochemicals such as phenolic acids and flavonoids, anthocyanins, dietary fibers, sorbitol, and minerals, particularly potassium, iron, magnesium, and calcium.^2−4^ The consumption of prunes has also been associated with preventing constipation,^5^ bone preservation,^6,7^ modulation of the immune response, reduced risk of diabetes, and progression of atherosclerosis.^8^

There have been many studies reporting changes in the phytochemical contents of prunes depending on the drying method, such as solar drying,^9^ freeze-drying,^9,10^ hot-air-drying,^9−13^ vacuum-drying,^10,14^ ultrasound-assisted osmotic dehydration,^15,16^ microwave-vacuum-drying,^14^ and microwave-freeze-drying.^17^ For example, a significant loss of neochlorogenic acid^9^ was reported in solar-dried prunes, whereas in hot-air-dried prunes, the chlorogenic acid loss^10^ was more significant. The hot-air-drying resulted in the greatest significant decrease in the anthocyanin pigment content (by an average of 82%), along with the total polyphenol content (by an average of 41%) and chlorogenic acid (an average of 69%), whereas the anthocyanin loss was an average of 11 and 10% in the vacuum- and freeze-dried prunes, respectively.^10^ It is crucial to choose the appropriate drying technique because it might negatively affect the nutritional value of the dried product.^2^

The phenolic substances should be released from food matrices during gastrointestinal digestion, become potentially available for absorption, and exert beneficial health effects on the human body.^18^ Previously, it was postulated that the microstructural changes that occurred during drying would affect the release of phenolics from food matrices during digestion and therefore their bioaccessibility.^19,20^ Drying has been shown to alter the cellular structure of apple,^21^ beetroot,^22^ persimmon,^23^ mushroom,^24^ and black Isabel grapes,^25^ as well as the release of phenolics and antioxidant activity in gastrointestinal fluids. Over the years, there have been some studies evaluating the effects of *in vitro* gastrointestinal (GI) digestion on the bioaccessibility of phenolics and antioxidant activities in plums.^26−30^ To the best of our knowledge, there have been no studies evaluating the *in vitro* bioaccessibility of phenolics and antioxidant activities of prunes dried by different methods. Therefore, in this study, hot-air-, vacuum-, ultrasound-assisted vacuum-, and freeze-dried prunes and fresh plums were evaluated in terms of their morphology, phenolic compounds, and antioxidant activities. In addition, the effects of *in vitro* gastrointestinal digestion on the antioxidant activities and phenolic compounds of the fresh and dried plums were assessed.

## 2 Materials and Methods

### 2.1 Chemicals and Reagents

ABTS (2,2-azino-bis(3-ethylbenzothiazoline)-6-sulfonic acid-diammonium salt, >98%), DPPH (2,2-diphenyl-1-picrylhydrazyl, 95%) radicals, Folin–Ciocalteu’s phenol reagent, neocuproine, Trolox (97%), 2,4,6-tris(2-pyridyl)-*s*-triazine (TPTZ), amylase (A1031), pepsin (P7012), pancreatin (P7545), bile (B3883), and analytical standards for HPLC were obtained from Sigma-Aldrich Ltd. (Steinheim, Germany).

### 2.2 Drying Procedure

European plum (*Prunus domestica* L.) samples were obtained from a local producer in August 2022 in Istanbul, Turkey. The fresh samples with bluish-black shells and juicy yellow flesh with uniform shape, size, color, and weight without visible surface damage or diseases selected for the experiments were washed with water, blotted gently, and stored at 4 °C for up to 12 h.

Plums as a whole were dried with four different methods; hot-air-drying (HAD) was done at 60 °C with a constant air velocity of 1.3 m/s (Memmert UF110, Germany). Vacuum-drying (VD) was performed in a vacuum drier (Daihan WOV30, South Korea), regulated by a vacuum pump (EVP 2XZ-2C, Zhejiang, China) with a 60 mbar pressure and 2 L/s speed. For ultrasound-assisted vacuum-drying (US-VD), the samples were placed into a conical flask connected to the vacuum pump and sonication was applied at 20 kHz by the ultrasonic water bath. Prefrozen samples (−80 °C) were dried by freeze-drying (FD) (Martin Christ, Germany). The drying time was 72, 20, 42, and 72 h for HAD, VD, US-VD, and FD, respectively. Each drying experiment was conducted in triplicate. The dried prunes were stored at 4 °C in sealed bags. The moisture content of the samples was 80.02 ± 0.20% (fresh), 26.15 ± 0.30% (HAD), 27.08 ± 0.12% (VD), 26.85 ± 0.43% (US-VD), and 26.03 ± 0.60% (FD).

### 2.3 Proximate Analysis of Plum

The proximate analysis of fresh plums, including ash, protein (977.02), fat (930.09), carbohydrate, and dietary fiber (991.43), was determined according to the Association of Official Analytical Chemists (AOAC) methods.^31^ The minerals were analyzed according to the AOAC method 999 × 10^31^ by atomic absorption spectrometry for the determination of different minerals.

### 2.4 Determination of Color and Rehydration Ratio

L* (whiteness/darkness), a* (redness/greenness), and b* (yellowness/blueness) values were measured by a chromameter (Konica Minolta CR-400, NJ). Two grams of the dried samples were immersed in 100 mL of distilled water at 25 and 50 °C for 420 min. At predetermined time intervals, the samples were taken out, blotted with tissue paper to eliminate the excess water on the surface, and weighed. The rehydration ratio (RR%) was calculated as
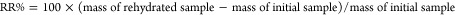


### 2.5 Scanning Electron Microscopy (SEM)

The samples were cut with a sharp razor blade, mounted on aluminum specimen stubs by using double-sided tape, and coated with a thin layer of gold film. The images were taken by an SEM (QUANTA FEG-250, FEI Company, OR) at 1500 and 2000 magnifications.

### 2.6 Preparation of Extracts

The plums (∼10 g of fresh or 2.5 g of dried samples) were homogenized with 80% of aqueous methanol (1:30, w:v) acidified with 0.1% HCl (v:v) at 10 000 rpm for 5 min (T25 Ultra-Turrax, IKA, NC), held on a magnetic stirrer overnight, and filtered. The residue was re-extracted twice, the combined extracts were centrifuged (2480*g*, 10 min, 4 °C), and the supernatant was evaporated under vacuum and then reconstituted to 6 mL with the extraction solvent and stored at −18 °C.

### 2.7 Total Phenolic, Total Flavonoid, and Total Anthocyanin Contents

The total phenolic content (TPC) of the samples was determined with the Folin–Ciocalteu (FC) reagent method according to the method described by Singleton and Rossi.^32^ An aliquot of 0.5 mL of the extracts was added to 2.5 mL of the FC reagent (0.2N) and 2 mL of a Na_2_CO_3_ (7.5%) solution. The mixture was incubated at room temperature for 30 min in the dark. The absorbance was measured at 760 nm using a Shimadzu 150 UV-1800 spectrophotometer (Kyoto, Japan).

The total flavonoid content (TFC) of the samples was determined according to the method described by Zhishen et al.^33^ The extract (1 mL) was mixed with 4 mL of distilled water, 0.3 mL of NaNO_2_ (5%), and 0.3 mL of an AlCl_3_ (10%) solution, and the mixture was left for 6 min. Then, 2 mL of NaOH (1 M) was added, and the volume was completed to 10 mL with distilled water. The absorbance was measured at 510 nm by a Shimadzu UV-1800 spectrophotometer.

The total monomeric anthocyanin (TA) content was determined according to the pH differential method of Giusti and Wrolstad.^34^ Absorbance was measured at 510 and 700 nm. The TA amount was determined by using the following equation
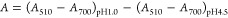

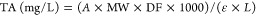
A is the absorbance at 510 and 700 nm, MW: molecular weight for cyanidin 3-O-glucoside (449.2 g/mol), ε: molar extinction coefficient for cyanidin 3-O-glucoside (26900), DF: dilution factor, and *L*: cell path length (1 cm).

The results of TPC, TFC, and TA were expressed as mg of gallic acid equivalent (GAE)/100 g of dw, mg of catechin equivalent (CE)/100 g of dw, and mg of cyanidin 3-O-glucoside (cy-3-O-glc)/100 g of dw, respectively.

### 2.8 Antioxidant Activity Assessment

The DPPH radical scavenging activity assay was carried out according to the method of Brand-Williams et al.^35^ Volumes of 4.9 mL of DPPH solution (0.1 Mm) and 0.1 mL of the extracts were mixed and incubated for 20 min in the dark at room temperature, and the absorbance was measured at 517 nm by a Shimadzu UV-1800 spectrophotometer. The cupric-reducing antioxidant capacity (CUPRAC) was performed as described by Apak et al.^36^ The 1 mL portions of CuCl_2_ (0.01 M), neocuproine (7.5 mM), and ammonium acetate buffer (1 M, pH 7.0) were mixed. After the addition of 0.1 mL of the extract and 1 mL of distilled water; the mixture was incubated at room temperature for 1 h in the dark. The absorbance was measured at 450 nm. ABTS radical scavenging activity was determined using the method described by Re et al.^37^ After adding 2 mL of diluted ABTS solution to a 0.1 mL extract, it was left in the dark for 6 min and the absorbance was measured at 734 nm. The FRAP (ferric reducing antioxidant power) assay was performed according to Benzie and Strain.^38^ A 100 μL portion of the extract was mixed with 900 μL of water and 2 mL of the FRAP reagent and incubated at room temperature for 30 min in the dark. The absorbance was measured at 593 nm. All results of antioxidant activity assessments were given as mmol of Trolox equivalent (TE)/100 g of dw.

### 2.9 HPLC Analysis of Phenolic Compounds

Phenolic compounds were determined using the HPLC system (LC-20AD pump, SIL-20A HT autosampler, CTO-10ASVP column oven, DGU-20A5R degasser, and CMB-20A communications bus module) coupled to a diode array detector—SPDM20A DAD (Shimadzu Corp., Japan) according to Karadag et al.^39^ Separations were conducted at 40 °C on an Inertrsil ODS C-18 reversed-phase column (250 mm × 4.6 mm, 5 μm particle size, GL Sciences, Japan). The individual anthocyanins were determined according to Capanoglu et al.^40^ The mobile phases were acetic acid in water (0.1:99, v:v) and acetic acid in acetonitrile (0.1:99, v:v). A gradient elution was applied as follows: 10% B (0–2 min), 10–30% B (2–27 min), 30–90% B (27–50 min), and 90–100% (51–60 min) and at 63 min returns to the initial conditions. Chromatograms were acquired at 278, 320, 360, and 520 nm to quantify phenolic acids, flavonoids, and anthocyanins. Identification and quantitative analyses were performed by comparing UV absorption spectra and retention times of each compound with those of external standards. The stock solutions of reference standards (gallic acid, caffeic acid, chlorogenic acid, neochlorogenic acid, ellagic acid, ferulic acid, rutin, quercetin, cyanidin 3-O-glucosides, and peonidin 3-O-glucosides) were prepared in methanol. The working solutions at concentrations ranging from 1 to 100 mg/L were achieved through the dilution of the stock solution with methanol. Calibration curves were generated by graphing the peak areas of the compounds identified relative to the peak areas against the concentration of the standard solution. The calibration curves based on duplicate injections demonstrated good linearity, with *R*^2^ values exceeding 0.99 (peak area vs concentration). All analyses were performed in triplicate, and the results were given as mg/100 g dw.

### 2.10 *In Vitro-*Simulated Digestion Procedure

*In vitro-*simulated digestion assay was performed according to Brodkorb et al.^41^ and Minekus et al.^42^ The fresh and dried plums were mixed 1:1 (w: (v) with simulated salivary fluid (SSF), α-amylase (75 U/mL), and CaCl_2_ (0.75 mM)) and vortexed for 2 min at 37 °C (pH 7.0). The oral bolus was diluted with simulated gastric fluid (SGF) (1:1, v:v), CaCl_2_ (0.075 mM), and pepsin (2000 U/mL) and incubated at 100 rpm for 2 h at 37 °C (pH 3.0). The gastric chyme was mixed with simulated intestinal fluid (SIF) (1:1, v:v), CaCl_2_ (0.3 mM), pancreatin (100 U/mL), and fresh bile (10 mM) at pH 7.0. The segments of dialysis bags (MWCO 12 kDa) filled with NaHCO_3_ (0.1 M) were placed in SIF medium, and the beakers were kept at 100 rpm for 2 h at 37 °C.^43^ The content of the dialysis bags was the compounds that entered the serum (IN), and those outside the bags were the material that remained in the GI tract (OUT). The supernatants taken for the oral, gastric, and intestinal phases were collected after centrifugation at 2480*g*, 10 min, 4 °C, filtered (0.45 μm), immediately frozen in liquid nitrogen, and lyophilized. The powders of lyophilized oral, gastric, and intestinal phases were redissolved in the extraction solvent (80% of aqueous methanol acidified with 0.1% HCl). A blank test tube without samples but with all digestion fluids was also subjected to analysis. The solid residue that remained after centrifugation at the end of intestinal digestion was collected as a nonbioaccessible fraction, and its methanolic extract was prepared (**2.6**). All procedures were performed in triplicate. Bioaccessibility index (BI%) and recovery percentage (*R*%) were calculated as^25,44^
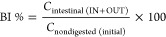

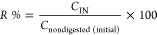


### 2.11 Statistical Analysis

All experiments were carried out in triplicate, and the data were reported as the mean ± the standard deviation. Statistical analysis was performed with SPSS Statistics Software (IBM version 20). One-way ANOVA Tukey’s post hoc test was applied to the responses (measured bioactive properties) to determine the differences among samples (the prunes obtained by four different drying methods and fresh plum).The differences in responses observed for each sample that was subjected to *in vitro* gastrointestinal digestion were compared among digestion phases. The calculated values of *R*% and BI% at the end of the simulated digestion phase were compared among the samples.^25^ Differences were considered significant if *p* < 0.05. A heat map was constructed with Origin Pro 2023 statistical analysis software (Origin Lab Corp., MA) to better illustrate the data (the change of individual phenolic compounds, TPC, TFA, TA, and antioxidant activities at each digestion stage).^45^

## 3 Results and Discussion

### 3.1 Proximate Composition of Fresh Plum

The dry matter content of fresh plum was 19.98 ± 0.2%, and the carbohydrate and dietary fiber contents were 16.90 ± 0.21 and 1.86 ± 0.05 g per 100 g of fresh plum, respectively. Plums were considered high-fiber fruits; the dietary fiber content was around 1.5 g per 100 g of fresh plums and can be elevated to 6.5 g per 100 g of dried fruits.^4^ Potassium (227.34 ± 2.96 mg/100 g) was the major mineral in our sample, followed by phosphorus, magnesium, and calcium (Table S1), and those levels were comparable to the previous studies.^8^

### 3.2 Physical Properties of Fresh and Dried Plums

In addition to being a quality indicator for consumer acceptance, the color of dried fruits was also associated with the content of anthocyanins and other phenolic compounds. The whiteness of prunes (L*) was increased by FD on the outer and inner surfaces; the other drying methods did not cause any considerable change in L* values on the outer surface but yielded darker colors on the inner surfaces (Table S2).

The increase in the lightness of freeze-dried fruits has been reported previously as related to the porous structure and existence of air voids and the difference in light diffusion that passes through the sample due to the replacement of water with air.^46,47^ The surface of fresh plum had purple tints with positive a* (redness) and negative b* (blueness) values, and those were generally reduced by drying, except for FD samples. US-VD yielded prunes with a more dull color and had the lowest a* and b* values. The color of the plum surface might also be related to the presence of anthocyanins, and the highest anthocyanin loss was determined in US-VD samples, followed by HAD (Table 1). The positive b* value (yellowness) observed on the inner surface of the fresh plum was significantly reduced by HAD and US-VD. On the contrary, compared to the fresh sample, similar to the lightness value, FD prunes presented a higher yellowness. The inner surface a* value of fresh plum was negative, indicating more green tints, and by drying, all samples presented positive a* values with hues of redness. The highest redness was observed in HAD and VD prunes, whereas in FD and US-VD samples, the a* value was similar. The increase in redness on the interior surface might be related to enzymatic and nonenzymatic reactions influenced by the presence of oxygen and the duration of heating. It has been reported that during hot-air-drying of plum varieties, the sucrose was hydrolyzed to reducing sugars that take part in Maillard browning reactions through the action of invertase and organic acids released due to the disruption of the cell membrane.^48^

**Table 1:** Total Phenolic Content (TPC), Total Flavonoid Content (TFC), Total Anthocyanin (TA), and Antioxidant Activities and Individual Phenolics of Fresh and Dried Plums^a^

	fresh	HAD	VD	US-VD	FD
TPC^b^	656.91 ± 17.49^b^	498.23 ± 23.11^c^	504.41 ± 58.78^c^	196.84 ± 7.27^d^	919.58 ± 28.81^a^
TFC^c^	247.10 ± 19.53^b^	276.53 ± 18.75^b^	172.00 ± 2.20^c^	39.56 ± 2.03^d^	432.34 ± 36.54^a^
TA^d^	50.28 ± 4.19^b^	1.71 ± 0.01^c^	59.47 ± 9.36^b^	0.72 ± 0.20^c^	87.01 ± 5.21^a^
Antioxidant Activity^e^ (mmol TE/100 g dw)					
DPPH	2.16 ± 0.10^b^	1.26 ± 0.03^c^	2.33 ± 0.19^b^	0.48 ± 0.01^d^	3.09 ± 0.05^a^
CUPRAC	5.39 ± 0.62^b^	3.58 ± 0.16^c^	4.34 ± 0.15^c^	1.06 ± 0.14^d^	7.66 ± 0.37^a^
ABTS	5.41 ± 0.54^b^	2.34 ± 0.27^c^	3.55 ± 0.07^c^	0.55 ± 0.05^d^	6.45 ± 0.08^a^
FRAP	2.84 ± 0.34^b^	1.93 ± 0.10^c^	2.94 ± 0.15^b^	0.44 ± 0.01^d^	4.06 ± 0.19^a^
Phenolics (mg/100 g dw)					
gallic acid	11.05 ± 1.15^a^	6.18 ± 0.76^b^	6.74 ± 0.01^b^	1.74 ± 0.01^c^	6.76 ± 0.17^b^
neochlorogenic acid	93.74 ± 6.47^b^	54.30 ± 1.85^c^	62.59 ± 2.58^c^	1.47 ± 0.10^d^	155.09 ± 0.23^a^
chlorogenic acid	51.13 ± 8.09^b^	32.55 ± 5.20^c^	24.29 ± 1.51^c^	0.98 ± 0.06^d^	80.34 ± 4.58^a^
caffeic acid	7.08 ± 0.29^a^	4.69 ± 0.01^b^	3.65 ± 0.00^c^	0.95 ± 0.20^d^	4.60 ± 0.23^b^
ellagic acid	13.38 ± 1.26^a^	6.94 ± 0.02^b^	7.65 ± 0.13^b^	1.56 ± 0.01^c^	7.63 ± 0.19^b^
ferulic acid	1.62 ± 0.01^a^	1.53 ± 0.02^b^	0.85 ± 0.00^d^	0.39 ± 0.00^e^	0.89 ± 0.00^c^
quercetin	28.99 ± 0.02^a^	15.87 ± 0.00^c^	15.93 ± 0.00^b^	nd	15.85 ± 0.00^c^
rutin	7.25 ± 0.74^b^	3.42 ± 0.07^c^	5.75 ± 0.24^c^	0.47 ± 0.02^d^	12.01 ± 1.16^a^
Anthocyanins (mg/100 g dw)					
Cyn-3-O-glc	34.45 ± 5.39^b^	nd	24.45 ± 0.28^c^	nd	45.14 ± 3.27^a^
Pn-3̅-O-glc	12.83 ± 1.61^a^	nd	8.17 ± 0.15^b^	nd	13.82 ± 1.94^a^

a Data are expressed as mean ± SD of triplicate measurements. Means with different letters in the same row are significantly different (*p* < 0.05). HAD, hot-air-drying; VD, vacuum-drying; US-VD, ultrasound-assisted vacuum-drying; FD, freeze-drying.

b TPC, total phenolic content (mg GAE/100 g dw).

c TFC, total flavonoid content (mg CE/100 g dw).

d TA; total anthocyanin content (mg cyanidin 3-O-glucoside (cyn-3-O-glc)/100 g dw).

e *DPPH*, 2,2-diphenyl-1-picrylhydrazyl radical scavenging activity; *CUPRAC*, copper reducing antioxidant capacity; *ABTS*, 2,2′-azino-bis (3-ethylbenzothiazoline)-6-sulfonic acid radical scavenging activity and *FRAP*, ferric reducing antioxidant power, *TE*, Trolox equivalent; Cyn-3-O-glc, cyanidin 3-O-glucoside; Pn-3̅-O-glc, peonidin 3-O-glucoside; nd, not detected.

The rehydration ratio is related to the extent of structural drying, and generally, a higher rehydration rate means less shrinkage and well-defined voids.^49,50^ FD samples showed the highest rehydration at both temperatures (25 and 50 °C), and the lowest ratio was observed in HAD and US-VD prunes (Figure S1). It is known that freeze-dried fruits have higher porosity and the least shrinkage, which would enhance water absorption.^47^ The lower rehydration of US-VD and HAD samples may indicate the presence of more shrunken and damaged cells and capillaries that lowered the intake of water. Additionally, the thick layer formed on the outer surface of plums by US-VD and HAD may also jeopardize the rehydration ratio.

### 3.3 Microstructural Changes

**Figure 1:**
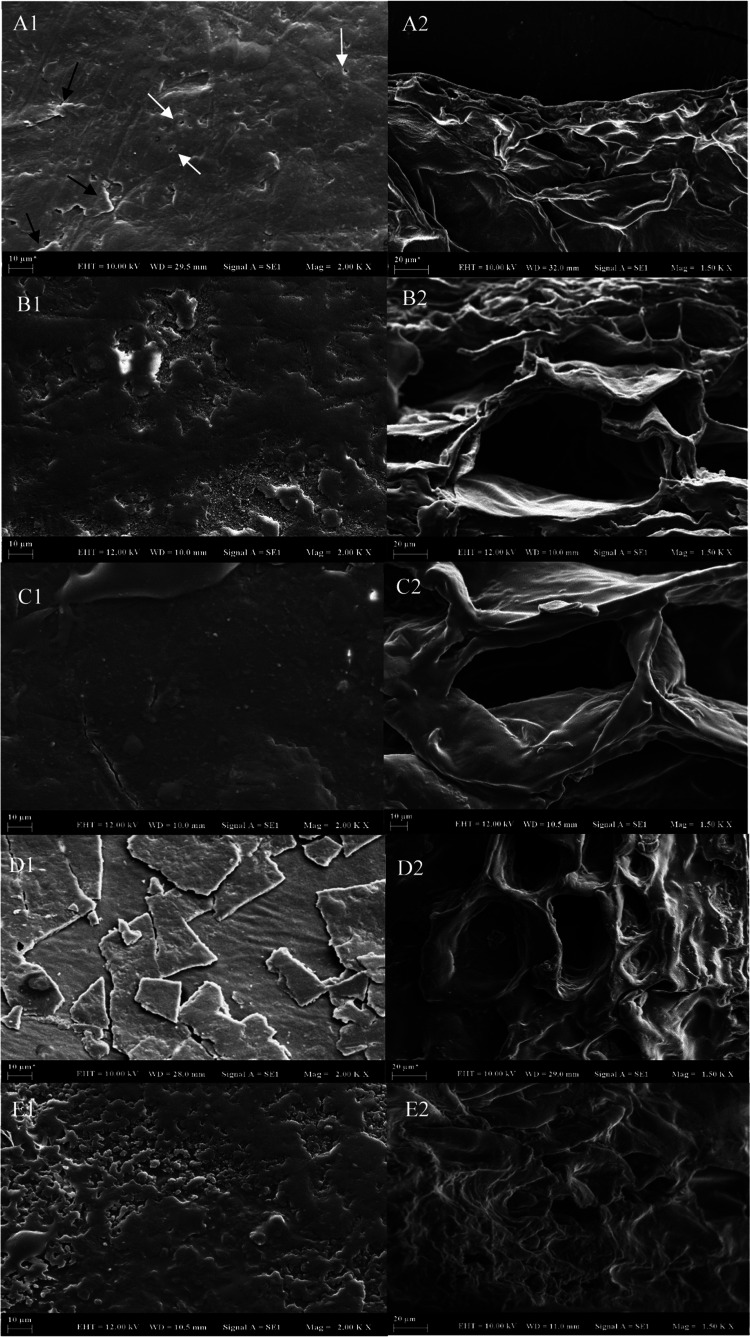
Morphology of fresh plum (A), prunes dried by FD (B), VD (C), HAD (D), and US-VD (E). FD, freeze-drying; VD, vacuum-drying; HAD, hot-air-drying; and US-VD, ultrasound-assisted vacuum-drying. 1 and 2 correspond to the outer surface (2000x magnification) and the cross section (1500× magnification) of the samples, respectively.

The surface of fresh plums (Figure 1A1) was smooth, and the presence of stomata (white arrows) that allow the passage of gas and the microwrinkles caused by the wax layer (black arrows) could be noticed. The cross section and the inner surface of the fresh plum (Figure 1A2) had larger parenchymal cells, showing no shrinkage and the presence of voids due to the high water content. Among the outer surfaces of dried plums, the ones obtained by FD (Figure 1B1) and VD (Figure 1C1) most resemble the surface of fresh plums, and the clustering of the wax layer due to dehydration and the presence of some cracks can be seen. The HAD plums (Figure 1D1) showed fractured surfaces with separate flaked layers. The drying of plums at high temperatures may cause the degradation of polysaccharides, alterations in the bonding between polymers, and the separation of the cells. In US-VD prunes (Figure 1E1), the surface had high levels of microgranular aggregates. In HAD and US-VD, the application of heat and ultrasound caused more microstructural stress due to the longer drying time and the application of ultrasonic cavitation. The cross-sectional images of dried samples presented more porous structures; however, their size, depth, and distribution varied by the methods of drying. In FD and VD (Figure 1B2,C2), larger hollow openings can be observed, whereas for HAD and especially US-VD samples, the voids and holes became shallow and distorted (Figure 1D2,E2). These results were also associated with the lower rehydration ratio of dried plums obtained by HAD and US-VD.

### 3.4 Total Phenol, Total Flavonoid Content, Antioxidant Activity, and Phenolic Composition of Fresh and Dried Plums

The total phenolic content (TPC) and total flavonoid content (TFC) of fresh plum were 656.91 ± 17.49 mg of GAE/100 g of dw and 247.10 ± 19.53 mg of CE/100 g of dw, respectively. Our results were in agreement with the results of Miletić et al.^51^ who determined the TPC of three blue-purple plum varieties between 215 and 643 mg GAE/100 g fw, and the TFC value ranged from 100 to 345 mg CE/100 g dw. Kaulmann et al.^52^ determined the TPC level in the range of 111–170 mg GAE/100 g and 27–76.5 mg CE/100 g dw in some European plum varieties (President, Italian, and Kirkes blue plums). The total anthocyanin content (TA) of our sample was 50.28 ± 4.19 mg cyn-3-O-glc/100 g dw, and it was determined between 5.59 and 12.8 mg cyn-3-O-glc/100 g fw in the similar plum varieties.^52^

Gościnna et al.^10^ studied the bioactive properties of three different blue-purple skin plums (Bluefree, Stanley, and Sweet Common) and when compared to our results, they found lower TPC values (173–225 mg GAE/g dw) but higher TA content (82–148.3 mg cyn-3-O-glc/100 g dw) in their three plum cultivars.

All drying methods, except for FD, caused a reduction in TPC levels compared with fresh samples. The highest reduction was observed under US-VD conditions, while the difference between HAD and VD was not significant. Similarly, US-VD-treated plums had the lowest TFC value, while HAD prunes had a higher amount of TFC, comparable to that of the fresh sample. The TA value of VD prunes was similar to that of fresh samples, whereas HAD and US-VD samples showed a dramatic decrease (around 98%). Our results were consistent with Gościnna et al.,^10^ who determined that HAD at 60 °C resulted in the greatest significant decrease in TA (by an average of 82%) and TPC (by an average of 41%). Reductions or losses of phenolic compounds by hot-air-drying have been associated with thermal and enzymatic degradation, especially when oxygen is present.^53^

The drying temperature in our study was 60 °C, and the drying time was in HAD (72 h), followed by that in US-VD (42 h) and VD (20 h). Although the drying time of HAD was higher than that of US-VD, the highest reduction of TPC, TFC, and TA was observed in US-VD. Due to the alternating compressions and expansions of the material during sonication, the water inside the material could move the surface through the microscopic channels; however, the crystalline waxy outer surface of the plum may not provide enough rubbery state to allow this water transfer to the surrounding environment and therefore could restrict the mobility of water.^54^ It may cause the creation of localized hotspot regions in the fruit that also trigger the loss of active compounds. The use of freeze-drying has been shown to significantly enhance the extractability of phenolics in blueberries,^55^ goji berries,^56^ and stinging nettle.^57^ The increase in the measured bioactive properties in FD prunes compared to that in fresh samples could be related to the enhanced extractability of those compounds into the extraction solvent due to the mild microstructural changes induced by the ice-crystal formation during freeze-drying and higher rehydration with the solvent due to the porous structure.

As shown in Table 1, the antioxidant activity of the samples followed a trend similar to the TPC, TFC, and TA values. The highest antioxidant activity values were observed in FD prunes, which were generally followed by fresh samples, and US-VD prunes displayed the lowest values. The difference between fresh and VD samples was not significantly different while evaluating antioxidant activity by FRAP and DPPH assays. The sum of all individual phenolics quantified by HPLC-DAD analysis was lower than the TPC value attained by the Folin–Ciocalteu spectrophotometric assay. Despite its popularity, the Folin–Ciocalteu test is not specifically designed for phenolic compounds, as the reagent could also be reduced by other nonphenolic compounds such as ascorbic acid, sugar, amino acid, and redox-active metal ions present in the sample, with the risk of content overestimation.^58^ HPLC analysis is more sensitive and specific to target phenolic compounds, and the quantification of individual phenolics was based on a comparison of the eluted peaks with the available reference standards.

Although they were not determined in our study, plums were reported to contain proanthocyanidin derivatives, such as the dimers procyanidin B1, B4, and B2. When five different plum cultivars were analyzed at different stages of maturation, although catechin or epicatechin was not detected in significant amounts, dimeric and trimeric forms of catechin were quantified, particularly in the skins of the fruit.^59^

In fresh plum, among phenolic acids, neochlorogenic acid (93.74 ± 6.47 mg/100 g dw) was the major compound, followed by chlorogenic acid (51.13 ± 8.09 mg/100 g dw), ellagic, caffeic, and gallic acids. Our results were consistent with Piga et al.^13^ as the neochlorogenic and chlorogenic acid contents in the Stanley cultivar were 381.77 and 54.51 mg/100 g dw, higher than the values determined by Gościnna et al.^10^ (111.7 mg and 42.5 mg/100 g dw, respectively). Kim et al.^60^ reported the contents of neochlorogenic acid (from 18.1 to 215.4 mg/100 g fw) and chlorogenic acid (0.9–21.0 mg/100 g fw) in the fresh fruits of 11 plum cultivars. The amount of chlorogenic and neochlorogenic acid contents was significantly reduced by drying, except for FD samples. The highest reduction (more than 95%) was observed in the US-VD prunes. HAD and VD drying resulted in a 33–42% reduction in neochlorogenic acid and up to a 50% reduction in the chlorogenic acid content. The amounts of those compounds determined in HAD and VD drying were not significantly different from each other. The decrease in the chlorogenic acid content could be due to the formation of neochlorogenic and cryptochlorogenic acid isomers, which occurred through isomerization due to molecular lactone migration at higher temperatures,^61^ whereas in FD prunes, a higher amount of chlorogenic acid derivatives was detected compared to the fresh sample. During FD, bioactive compounds are expelled from the epithelial cells at a higher rate, and the release of polyphenol compounds might be increased due to the breakdown of cellular constituents.^62^ Among flavonoids, rutin (quercetin 3-rutinoside) was determined in our samples.^13,51,60^ The rutin content of fresh plums was significantly higher than that of dried samples except FD prunes. The presence of anthocyanins such as cyanidin 3-O-glucoside, cyanidin 3-O-rutinoside, peonidin 3-O-glucoside, and peonidin 3-O-rutinoside^10,60,63^ in plums has been previously reported. By comparison with the available external standards we had, cyanidin 3-O-glucoside (34.45 ± 5.39 mg/100 g of dw) and peonidin 3-O-glucoside (12.83 ± 1.61 mg/100 g of dw) were detected and quantified in our sample (Table 1). Only FD and VD samples had anthocyanin constituents, and compared to fresh samples, their level was reduced in VD prunes (29–36% reduction).

### 3.5 Effect of Drying Methods on the *In Vitro* Bioaccessibility of Plum Phenolics and Antioxidant Activity

The change in TPC, TFC, TA, and antioxidant activity values at various stages of *in vitro* gastrointestinal digestion, including the oral, gastric, and intestinal phases, was given in Table 2. TPC, TFC, and TA levels in the oral phase were lower compared with the initial levels found in the methanolic extract (Table 1) of undigested samples. This decrease can be attributed to the limited solubility of these compounds in simulated saliva fluid and the relatively short duration of this step (2 min). Among the dried plums, FD prunes exhibited the highest release of TPC and TFC, potentially due to their porous structure, which could enhance extractability in the oral bolus. Generally, in all samples, TPC, TFC, CUPRAC, ABTS, and FRAP values increased at the gastric phase. In previous studies, an increase in the amount of biologically accessible polyphenols and flavonoids has been observed during gastric digestion.^28,64−66^ The release of polyphenols from apples after simulated gastrointestinal digestion was mainly achieved in the gastric phase. However, their content was still significantly lower than that obtained by the methanol extraction.^67^ Although it was reported that most of the phenolic compounds in plum fruits were present in free form, the ratio of free phenolics to the total phenolic content (free+bound) was reported to be around 70% in Natal plum,^30^ 92%,^29^ and 80–90%^27^ in Japanese plum varieties. When Seke et al.^30^ studied the effect of free and bound phenolic compounds on the antioxidant activity of plum fruit, they concluded that although most of the phenolic compounds were present in free form (939.03 mg GAE/kg fw), TPC in the bound insoluble fraction of plum fruits was higher after acid hydrolysis (255.5 mg GAE/kg fw) than after alkaline hydrolysis (137.13 mg GAE/kg fw). Therefore, the longer incubation time of the gastric stage could lead to a higher release of free phenolics, and the acidic medium and the presence of digestive enzymes may also facilitate the release of some bound phenolics.^68^

**Table 2:** Change in the Total Phenolic Content (TPC), Total Flavonoid Content (TFC), Total Anthocyanin (TA), and Antioxidant Activities of Fresh and Dried Plums during *In Vitro* Digestion^a^,^b^

			intestinal			
	oral	gastric	IN	OUT	*R*%	BI %
TPC						
fresh	88.04 ± 7.51^c^	349.03 ± 45.78^a^	17.93 ± 2.49^d^	193.11 ± 22.03^b^	2.72 ± 0.32^B^	32.19 ± 3.93^C^
HAD	103.18 ± 7.78^b^	230.29 ± 18.84^a^	22.65 ± 2.59^c^	235.64 ± 1.71^a^	4.53 ± 0.36^A^	51.89 ± 1.85^B^
VD	86.36 ± 5.98^b^	132.76 ± 14.10^a^	2.37 ± 0.40^c^	62.60 ± 5.89^b^	0.48 ± 0.13^C^	13.23 ± 4.49^D^
US-VD	24.78 ± 2.74^c^	142.75 ± 6.92^b^	5.30 ± 1.54^c^	217.31 ± 17.97^a^	2.71 ± 0.84^B^	113.01 ± 4.90^A^
FD	139.80 ± 15.13^c^	355.35 ± 26.57^a^	17.35 ± 1.44^d^	253.27 ± 65.34^b^	1.89 ± 0.21^B^	29.50 ± 7.66^C^
TFC						
fresh	22.01 ± 2.10^b^	281.18 ± 3.02^a^	3.49 ± 1.04^c^	5.62 ± 2.47^c^	1.41 ± 0.38^B^	3.64 ± 1.09^B^
HAD	29.77 ± 1.32^b^	68.07 ± 0.33^a^	2.39 ± 0.38^d^	11.50 ± 3.22^c^	0.86 ± 0.08^BC^	4.99 ± 1.00^B^
VD	20.66 ± 1.02^b^	37.38 ± 3.01^a^	1.35 ± 0.69^d^	12.93 ± 0.81^c^	0.78 ± 0.39^BC^	8.30 ± 0.39^B^
US-VD	7.52 ± 1.48^b^	33.80 ± 2.93^a^	1.11 ± 0.10^c^	5.13 ± 1.33^bc^	2.83 ± 0.31^A^	15.95 ± 4.23^A^
FD	59.82 ± 1.01^c^	243.46 ± 7.27^a^	2.67 ± 0.17^d^	88.72 ± 2.65^b^	0.61 ± 0.01^C^	21.20 ± 1.24^A^
TA						
fresh	5.76 ± 2.38^b^	16.40 ± 3.62^a^	0.33 ± 0.08^b^	1.73 ± 0.57^b^	0.67 ± 0.21^B^	4.19 ± 1.59^B^
HAD	0.09 ± 0.02^b^		0.13 ± 0.04^b^	0.42 ± 0.13^a^	8.17 ± 2.72^B^	33.30 ± 7.08^B^
VD	1.69 ± 0.62^ab^	2.55 ± 0.38^a^	0.01 ± 0.00^c^	1.49 ± 0.08^b^	0.02 ± 0.00^B^	2.59 ± 0.56^B^
US-VD	1.06 ± 0.16^a^	0.10 ± 0.04^c^	0.12 ± 0.01^c^	0.61 ± 0.10^b^	18.49 ± 6.42^A^	109.15 ± 38.41^A^
FD	2.08 ± 1.17^b^	28.95 ± 8.46^a^	0.13 ± 0.02^b^	2.45 ± 0.44^b^	0.11 ± 0.09^B^	2.98 ± 0.57^B^
DPPH						
fresh	370.16 ± 46.25^a^	207.66 ± 15.06^b^	115.60 ± 35.25^c^	51.83 ± 12.58^c^	5.32 ± 1.47^A^	7.74 ± 1.34^C^
HAD	130.73 ± 34.07^b^	185.89 ± 6.53^a^	50.71 ± 2.28^c^	88.63 ± 6.74^bc^	4.02 ± 0.07^AB^	11.05 ± 0.42^B^
VD	171.96 ± 49.83^a^	73.79 ± 9.90^b^	14.68 ± 1.94^b^	59.40 ± 19.37^b^	0.63 ± 0.07^C^	3.22 ± 1.01^D^
US-VD	108.52 ± 10.96^a^	16.81 ± 8.56^c^	9.86 ± 3.13^c^	67.42 ± 9.70^b^	2.03 ± 0.59^BC^	16.01 ± 1.68^A^
FD	250.01 ± 19.10^b^	1830.10 ± 34.24^a^	121.39 ± 17.75^c^	72.46 ± 12.10^c^	3.92 ± 0.57^AB^	6.27 ± 0.52^C^
CUPRAF						
fresh	668.58 ± 33.57^c^	1750.25 ± 108.02^a^	177.65 ± 24.10^d^	856.91 ± 51.07^b^	3.30 ± 0.44^B^	19.35 ± 2.81^C^
HAD	558.56 ± 56.06^b^	1387.32 ± 53.82^a^	93.32 ± 15.22^c^	677.28 ± 100.33^b^	2.61 ± 0.50^B^	21.48 ± 1.46^C^
VD	590.52 ± 58.95^b^	617.87 ± 2.46^b^	30.17 ± 6.38^c^	831.39 ± 111.35^a^	0.69 ± 0.14^C^	19.80 ± 2.09^C^
US-VD	243.26 ± 48.76^b^	681.62 ± 86.78^a^	66.50 ± 3.81^c^	585.73 ± 48.28^a^	6.30 ± 0.58^A^	61.94 ± 8.09^A^
FD	901.02 ± 46.07^b^	3592.00 ± 247.93^a^	177.30 ± 15.77^c^	3156.6 ± 416.92^a^	2.31 ± 0.10^B^	43.48 ± 4.34^B^
ABTS						
fresh	378.14 ± 95.78^bc^	3856.29 ± 241.48^a^	142.66 ± 20.00^c^	569.70 ± 78.04^b^	2.65 ± 0.47^BC^	13.21 ± 2.50^BC^
HAD	195.28 ± 58.99^b^	530.79 ± 12.20^a^	44.91 ± 24.32^c^	76.30 ± 10.69^c^	1.93 ± 0.37^BC^	5.21 ± 0.56^C^
VD	200.00 ± 19.01^b^	396.85 ± 16.69^a^	107.03 ± 20.53^c^	453.77 ± 37.57^a^	3.01 ± 0.63^B^	15.77 ± 1.23^BC^
US-VD	59.05 ± 8.91^c^	368.84 ± 54.58^a^	72.38 ± 7.36^c^	204.75 ± 37.99^b^	12.96 ± 0.77^A^	50.95 ± 9.74^A^
FD	344.59 ± 196.5^b^	1549.67 ± 15.01^a^	94.49 ± 19.53^b^	1345.91 ± 298.9^a^	1.46 ± 0.32^C^	22.30 ± 4.61^B^
FRAP						
fresh	261.97 ± 0.96^b^	3011.38 ± 100.46^a^	55.01 ± 4.64^c^	215.02 ± 2.54^b^	1.97 ± 0.37^C^	9.62 ± 1.21^C^
HAD	198.66 ± 32.61^b^	430.78 ± 36.14^a^	107.10 ± 1.04^b^	438.43 ± 101.13^a^	5.55 ± 0.34^A^	28.46 ± 6.76^B^
VD	127.25 ± 3.98^b^	414.80 ± 15.20^a^	4.11 ± 0.20^d^	94.07 ± 9.57^c^	0.14 ± 0.01^D^	3.34 ± 0.22^C^
US-VD	120.89 ± 8.09^b^	466.19 ± 27.72^a^	11.80 ± 1.04^c^	90.82 ± 14.83^b^	2.70 ± 0.26^B^	23.53 ± 3.11^B^
FD	514.49 ± 26.33^c^	2734.13 ± 43.09^a^	113.26 ± 5.57^d^	1590.71 ± 130.5^b^	2.78 ± 0.08^B^	42.08 ± 4.55^A^

a Data are expressed as mean ± SD of triplicate measurements. Different lowercase letters in the same row for each sample are significantly different (*p* < 0.05) among digestion phases. Different capital letters on the same column for *R*% and BI% are significantly different (*p* < 0.05) among prune samples. *R*% (recovery percent) = *C*_IN_/*C*_nondigested_ × 100 BI%, (bioaccessibility index) = *C*_intestinal_ (IN + OUT)/*C*_nondigested_.

b HAD, hot-air-drying; VD, vacuum-drying; US-VD, ultrasound-assisted vacuum-drying; FD, freeze-drying; TPC, total phenolic content (mg GAE/100 g dw); TFC, total flavonoid content (mg CE/100 g dw); TA, total monomeric anthocyanins (mg cyn-3-O-glc/100 g dw); *DPPH*, 2,2-diphenyl-1-picrylhydrazyl radical scavenging activity; *CUPRAC*, copper reducing antioxidant capacity; *ABTS*, 2,2′-azino-bis (3-ethylbenzothiazoline)-6-sulfonic acid radical scavenging activity; and *FRAP*, ferric reducing antioxidant power (μmol TE (Trolox equivalent)/100 g dw).

The sum of the OUT+IN portion represents the entire intestinal phase. Compared to the gastric step of digestion, after the intestinal stage (IN+OUT), the TPC value was only increased in HAD and US-VD samples.^25^ The FC reagent of the TPC assay could also give positive results with the Maillard reaction products;^69^ therefore, due to the longer drying time, the HAD and US-VD prunes could have Maillard reaction products that could be available in the intestinal digestive slurry and present higher TPC values. The bioaccessibility (BI, %) of TPC in our plum samples ranged from 13.23% (VD) to 113.01% (US-VD) (Table 2). The BI (%) of TPC from pomegranate arils was reported to change from 50.61 (fresh) to 97.25% (US-VD).^70^ Compared to the gastric stage, there was a significant decrease in the TFC and TA values in all samples due to intestinal digestion conditions. The loss of TFC and TA was associated with their instability under the alkaline conditions of the small intestine, which could be related to the oxidation and polymerization reactions they undergo, the formation of higher-molecular-weight phenolic derivatives with low solubility, and different chemical properties.^24,29^ The bioaccessibility (%) of TFC was highest in FD (21.20%) and US-VD (15.95%) and lowest in fresh (3.64%) samples (Table 2). Although the US-VD sample has the highest bioaccessibility of TPC, TA, CUPRAC, and ABTS values, it is mostly related to the lower initial level of the measured properties in the extract and not because of the highest value determined after digestion.

The antioxidant activity of the prune samples was examined by DPPH, CUPRAC, ABTS, and FRAP assays. The alteration in the antioxidant activity values of digested samples did not exhibit a consistent pattern across all assays. As a parallel with the TPC value, except for the DPPH assay, the antioxidant activities increased after the gastric phase and decreased in the intestinal phase. It could be related to the assay conditions; only the DPPH assay has a radical soluble in the organic solvent, whereas the other assays were all conducted in an aqueous environment. As in our work, ABTS and hydroxyl radical scavenging assays showed that the gastric digestion products of mulberry fruits had the highest antioxidant activity when compared to intestinal samples.^71^ ABTS radical scavenging activities decreased by 36% and FRAP decreased by 40% during the intestinal degradation of red cabbage. This reduction in the parameters could be due to unidentified chemical transformations of the phenolic compounds, in particular, of the acylated anthocyanins.^72^

The residues of the samples after the small intestinal digestion were extracted and analyzed the same way as for the undigested samples. The TPC, TFC, TA, and antioxidant activity values yielded by the digested residues were quite low compared to those obtained before digestion (Table 3). The recovery values of TPC in those digested residues were changed from 1.18 to 14.50% in dried samples; the lowest was determined in FD, and the highest was in HAD and VD samples, while it was 3.47% in fresh plum. In the previous studies conducted on grape samples, it was also indicated that a significant portion of phenolics was extracted from the digested fractions.^25,44^

**Table 3:** Total Phenolic Content (TPC), Total Flavonoid Content (TFC), Total Anthocyanin (TA), and Antioxidant Activities and Individual Phenolics of Nonbioaccessible Fraction—the Digested Residue Remained after *In Vitro* Digestion^a^

	fresh	HAD	VD	US-VD	FD
TPC^b^	22.82 ± 2.27^b^	72.23 ± 9.35^a^	72.19 ± 8.69^a^	14.52 ± 0.19^b^	10.92 ± 1.16 ^b^
	*3.47%*	*14.50%*	*14.30%*	*7.37%*	*1.18%*
TFC^c^	0.93 ± 0.53^c^	3.39 ± 0.47^b^	4.57 ± 0.40^ab^	0.96 ± 0.24^c^	5.43 ± 0.80^a^
	*0.37%*	*1.22%*	*2.68%*	*2.42%*	*1.25%*
TA^e^	0.96 ± 0.28^a^				0.85 ± 0.12^a^
	*1.94%*				*0.97%*
Antioxidant Activity^d^ (μmol TE/100 g dw)					
DPPH	14.60 ± 7.39^c^	57.28 ± 22.25^ab^	20.16 ± 1.25^c^	27.21 ± 7.57^bc^	69.61 ± 13.36^a^
	*0.67%*	4.54*%*	*0.86%*	*5.66%*	*2.25%*
CUPRAC	97.98 ± 15.70^b^	153.20 ± 4.76^a^	147.98 ± 13.15^a^	163.99 ± 22.86^a^	152.91 ± 1.88^a^
	*1.81%*	*4.27%*	*3.40%*	*15.47%*	*1.99%*
ABTS	53.37 ± 5.82^a^	13.83 ± 1.41^b^	11.76 ± 1.31^b^	21.53 ± 6.09^b^	12.06 ± 0.38^b^
	*0.98%*	*0.59%*	*0.33%*	*3.98%*	*0.18%*
FRAP	30.68 ± 0.76^c^	194.10 ± 5.47^b^	49.77 ± 0.28^c^	37.14 ± 1.49^c^	241.14 ± 15.87^a^
	*1.08%*	*10.05%*	*1.69%*	8.44*%*	*5.93%*
Phenolics (mg/100 g dw)					
gallic acid	1.06 ± 0.01^a^	0.39 ± 0.01^c^	0.40 ± 0.01^c^	0.54 ± 0.01^b^	0.31 ± 0.02^d^
	*9.59%*	*6.31%*	*5.93%*	*31.03%*	*4.58%*
neochlorogenic acid					
chlorogenic acid	0.12 ± 0.00^c^	0.46 ± 0.13^a^	0.25 ± 0.05^ab^	0.23 ± 0.00^ab^	0.35 ± 0.14^ab^
	*0.23%*	*1.43%*	*1.02%*	*23.71%*	*0.43%*
caffeic acid		0.17 ± 0.00^b^	0.14 ± 0.00^c^	0.20 ± 0.01^a^	0.18 ± 0.02^b^
		*3.62%*	*3.83%*	*21.27%*	*3.91%*
ellagic acid		0.24 ± 0.00^b^	0.28 ± 0.00^b^		0.38 ± 0.04^a^
		*3.45%*	*3.66%*		*4.98%*
ferulic acid		0.11 ± 0.00^a^	0.06 ± 0.00^b^	0.11 ± 0.00^a^	0.04 ± 0.00^b^
		7.18%	*7.05%*	*28.20%*	*4.49%*
quercetin		0.63 ± 0.03^b^	0.65 ± 0.02^b^		0.63 ± 0.05^b^
		*3.96%*	*4.08%*		*3.97%*
rutin		0.05 ± 0.03^c^	0.19 ± 0.06^b^	0.07 ± 0.00^c^	1.02 ± 0.02^a^
		*1.46%*	*3.86%*	*14.89%*	*8.49%*

a Data are expressed as mean ± SD of triplicate measurements. The percentage values (%) indicate the ratio of the measured value remained in the digested residue to the value of the undigested sample. Means with different letters in the same row are significantly different (*p* < 0.05). HAD, hot-air-drying; VD, vacuum-drying; US-VD, ultrasound-assisted vacuum-drying; and FD, freeze-drying.

b TPC, total phenolic content (mg GAE/100 g dw).

c TFC, total flavonoid contents (mg CE/100 g dw).

d *DPPH*, 2,2-diphenyl-1-picrylhydrazyl radical scavenging activity; *CUPRAC*, copper reducing antioxidant capacity; *ABTS*, 2,2′-azino-bis (3-ethylbenzothiazoline)-6-sulfonic acid radical scavenging activity, *FRAP*, ferric reducing antioxidant power, *TE*, Trolox equivalent.

e TA, total monomeric anthocyanins (mg cyn-3-O-glc/100 g dw).

### 3.6 Changes in the Levels of Individual Phenolics of Fresh and Dried Plums during *In Vitro* Digestion

The change of individual phenolics and anthocyanins (mg/100 g dw) upon digestion is given in Table 4. As expected in all samples, the amount of individual phenolics increased from the oral to the gastric phase.^73^ Compared to the previous digestion step, after intestinal digestion (IN+OUT), the amount of neochlorogenic, chlorogenic, and caffeic acids decreased in all samples, while the amount of other determined compounds (gallic acid, ellagic acid, ferulic acid, and rutin) either decreased or increased depending on the samples. Cyn-3-O-glc and pn-3-O-glc were only detected in the gastric phase of fresh, VD, and FD samples; no anthocyanins were recovered after intestinal digestion. During the *in vitro* digestion of fresh and sun-dried figs, all phenolic acids and anthocyanins decreased by both drying and digestion.^19^ Anthocyanins cannot maintain their stability due to the high pH when passing through the gastrointestinal tract and the increase in ambient temperature during drying processes.^74^ It was also reported that the decrease in the bioaccessibility of anthocyanins could be related to their transformation into other phenolic substances during *in vitro* digestion, for example, cyn-3-O-glc and cyn-3-O-rut could be converted to cyanidin aglycones, ferulic acid, and caffeic acids.^75^

**Table 4:** Change in the Individual Phenolics and Anthocyanins of Fresh and Dried Plums during *In Vitro* Digestion^a^^b^

		oral	gastric	intestinal		*R*%	BI %
				IN	OUT		
Phenolics (mg100/g dw)							
gallic acid	fresh	1.68 ± 0.00^ab^	2.20 ± 0.05^a^	0.95 ± 0.05^b^	2.39 ± 0.60^a^	8.65 ± 0.40^B^	30.85 ± 8.23^B^
	HAD	0.80 ± 0.02^b^	1.68 ± 0.05^a^	0.28 ± 0.00^c^	1.67 ± 0.01^a^	4.63 ± 0.66^C^	32.02 ± 4.15^B^
	VD	0.59 ± 0.01^b^	0.55 ± 0.00^b^	0.27 ± 0.00^c^	1.08 ± 0.06^a^	4.03 ± 0.01^C^	20.05 ± 0.99^B^
	US-VD	0.88 ± 0.05^b^	1.24 ± 0.00^a^	0.43 ± 0.02^c^	1.33 ± 0.26^a^	24.64 ± 1.07^A^	100.92 ± 16.04^A^
	FD	0.60 ± 0.02^c^	1.37 ± 0.03^a^	0.30 ± 0.05^d^	0.86 ± 0.07^b^	4.47 ± 0.72^C^	17.26 ± 1.54^B^
neochlorogenic acid	fresh	3.03 ± 0.08^b^	77.13 ± 7.40^a^	0.04 ± 0.01^b^	0.57 ± 0.00^b^	0.04 ± 0.01^A^	0.65 ± 0.01^D^
	HAD	7.03 ± 0.72^b^	24.17 ± 0.74^a^	nd	1.35 ± 0.04^c^		2.49 ± 0.08^C^
	VD	0.10 ± 0.01^c^	0.34 ± 0.04^b^	nd	0.44 ± 0.02^a^		0.71 ± 0.02^D^
	US-VD	0.18 ± 0.01^c^	0.55 ± 0.00^b^	nd	0.81 ± 0.01^a^		54.89 ± 0.53^A^
	FD	16.81 ± 1.42^b^	78.21 ± 0.68^a^	nd	8.67 ± 1.27^c^		5.59 ± 0.82^B^
chlorogenic acid	Fresh	5.65 ± 0.12^b^	41.24 ± 4.02^a^	1.65 ± 0.03^bc^	0.22 ± 0.01^d^	3.29 ± 0.44^B^	3.74 ± 0.50^C^
	HAD	8.49 ± 0.68^b^	16.57 ± 0.98^a^	0.96 ± 0.10^d^	6.04 ± 0.74^c^	3.05 ± 0.82^BC^	22.20 ± 6.23^B^
	VD	0.18 ± 0.00^c^	1.22 ± 0.08^a^	0.08 ± 0.01^c^	0.37 ± 0.03^b^	0.34 ± 0.08^C^	1.90 ± 0.02^C^
	US-VD	0.19 ± 0.06^c^	4.07 ± 0.01^a^	0.08 ± 0.02^d^	0.35 ± 0.00^b^	8.76 ± 2.06^A^	45.58 ± 0.36^A^
	FD	9.69 ± 0.42^c^	40.38 ± 2.17^a^	1.58 ± 0.12^d^	34.42 ± 2.30^b^	1.97 ± 0.27^BC^	45.02 ± 5.59^A^
caffeic acid	fresh	1.11 ± 0.08^b^	2.54 ± 0.11^a^	0.55 ± 0.01^d^	0.87 ± 0.00^c^	7.84 ± 0.25^B^	20.29 ± 0.77^C^
	HAD	1.29 ± 0.23^b^	2.59 ± 0.08^a^	0.17 ± 0.00^d^	0.58 ± 0.03^c^	3.66 ± 0.11^B^	16.18 ± 0.80^C^
	VD	0.63 ± 0.13^b^	1.53 ± 0.05^a^	0.14 ± 0.00^c^	0.29 ± 0.00^c^	3.93 ± 0,01^B^	11.86 ± 0.13^C^
	US-VD	0.41 ± 0.01^b^	1.26 ± 0.03^a^	0.23 ± 0.01^b^	0.44 ± 0.01^b^	25.15 ± 4.23^A^	73.05 ± 15.58^A^
	FD	1.07 ± 0.04^c^	3.88 ± 0.07^a^	0.20 ± 0.01^d^	1.80 ± 0.01^b^	4.37 ± 0.18^B^	43.69 ± 2.07^B^
ellagic acid	fresh	0.80 ± 0.80^b^	2.52 ± 0.07^a^	1.06 ± 0.16^b^	1.82 ± 0.04^ab^	8.04 ± 1.83^A^	21.81 ± 3.29^B^
	HAD	0.73 ± 0.06^b^	2.80 ± 0.46^a^	0.37 ± 0.03^b^	0.50 ± 0.02^b^	5.42 ± 0.48^B^	12.66 ± 0.81^C^
	VD	0.88 ± 0.00^b^	1.57 ± 0.03^a^	nd	0.52 ± 0.01^c^		6.83 ± 0.07^C^
	US-VD	0.87 ± 0.07^b^	1.19 ± 0.02^a^	nd	0.94 ± 0.03^b^		59.99 ± 1.34^A^
	FD	1.33 ± 0.01^a^	1.55 ± 0.05^a^	0.70 ± 0.01^b^	1.55 ± 0.38^a^	9.22 ± 0.43^A^	29.62 ± 5.93^B^
ferulic acid	fresh	0.21 ± 0.00^c^	0.34 ± 0.01^a^	0.12 ± 0.00^d^	0.26 ± 0.00^b^	7.95 ± 0.07^B^	24.38 ± 0.54^C^
	HAD	0.15 ± 0.04^c^	0.50 ± 0.00^a^	0.04 ± 0.00^c^	0.29 ± 0.08^d^	2.82 ± 0.42^B^	22.41 ± 6.56^C^
	VD	0.08 ± 0.01^b^	0.29 ± 0.00^a^	0.03 ± 0.00^c^	0.10 ± 0.00^b^	3.88 ± 0.65^B^	15.95 ± 1.29^C^
	US-VD	0.13 ± 0.01^b^	0.26 ± 0.09^a^	0.07 ± 0.00^b^	0.09 ± 0.00^b^	17.50 ± 4.33^A^	40.00 ± 4.33^B^
	FD	0.15 ± 0.00^c^	0.74 ± 0.03^b^	0.03 ± 0.005^d^	1.18 ± 0.03^a^	4.44 ± 0.00^B^	135.55 ± 4.44^A^
quercetin	fresh	nd	3.85 ± 0.00^a^	nd	3.32 ± 0.52^a^		11.45 ± 0.04^A^
	HAD	1.05 ± 0.00^a^	1.05 ± 0.001^a^	nd	1.05 ± 0.00^a^		6.62 ± 0.08^B^
	VD	1.04 ± 0.00^b^	1.05 ± 0.00^ab^	nd	1.06 ± 0.00^a^		6.65 ± 0.06^B^
	US-VD	nd		nd	nd		
	FD	1.06 ± 0.00^ab^	1.05 ± 0.001^b^	0.63 ± 0.00^c^	1.06 ± 0.00^a^	3.99 ± 0.00^A^	6.69 ± 0.13^B^
rutin	fresh	0.45 ± 0.04^b^	14.90 ± 1.87^a^	0.64 ± 0.04^b^	0.86 ± 0.10^b^	8.93 ± 0.22^A^	20.96 ± 0.06^C^
	HAD	1.78 ± 0.24^c^	4.87 ± 0.10^a^	0.22 ± 0.00^d^	2.77 ± 0.17^b^	6.45 ± 0.43^B^	87.58 ± 3.04^AB^
	VD	1.42 ± 0.41^b^	3.13 ± 0.10^a^	0.04 ± 0.00^c^	0.18 ± 0.02^c^	0.83 ± 0.13^D^	4.10 ± 0.43^C^
	US-VD	0.19 ± 0.07^bc^	2.53 ± 0.11^a^	0.04 ± 0.00^c^	0.31 ± 0.08^b^	10.43 ± 1.55^A^	76.36 ± 16.01^B^
	FD	3.97 ± 0.01^c^	10.32 ± 0.24^b^	0.51 ± 0.03^d^	12.01 ± 1.16^a^	4.30 ± 0.12^C^	104.30 ± 0.12^A^
Anthocyanins (mg/100 g dw)							
Cyn-3-O-glc	fresh	nd	17.76 ± 0.35	nd	nd		
	HAD	nd	nd	nd	nd		
	VD	nd	0.58 ± 0.02	nd	nd		
	US-VD	nd	nd	nd	nd		
	FD	nd	19.40 ± 1.96	nd	nd		
Pn-3̅-O-glc	fresh	nd	6.83 ± 1.25	nd	nd		
	HAD	nd	nd	nd	nd		
	VD	nd	nd	nd	nd		
	US-VD	nd	nd	nd	nd		
	FD	nd	6.25 ± 0.23	nd	nd		

a Data are expressed as mean ± SD of triplicate measurements. Different lowercase letters in the same row for each sample are significantly different (*p* < 0.05) among digestion phases. Different capital letters on the same column for RI% and BI% are significantly different (*p* < 0.05) among prune samples. % (recovery percent) = *C*_IN_/*C*_nondigested_ × 100 BI%, (bioaccessibility index) = *C*_intestinal_ (IN+OUT)/*C*_nondigested_ cyn-3-O-glc: cyanidin 3-O-glucoside, Pn3̅-O-glc: peonidin 3-O-glucoside. nd: not detected.

b HAD, hot-air-drying; VD, vacuum-drying; US-VD, ultrasound-assisted vacuum-drying; and FD, freeze-drying.

Both the amount of individual substances (Table 4) and other measured properties (Table 2) passing into the IN medium through the dialysis tube have generally been lower. The possible formation of compounds with high molecular weight in the intestinal phase due to polymerization reactions could reduce the amount passing through to the IN phase. Compared to the gastric stage, the loss of stability of phenolic compounds in intestinal fluid, oxidation, and polymerization of phenolic compounds at high pH could be the reason for the reduced level of individual phenolics in the intestinal phase.^64,76^ In the previous studies,^29,30^ the main phenolic compounds determined in the bound fraction of plum fruit were reported to be catechin, epicatechin, caffeic acid, protocatechuic acid, ellagic acid, and ferulic acid. While Yu et al.^29^ did not determine chlorogenic and neochlorogenic acids, the main phenolic acids of plums, in the free phenolic fraction, Seke et al.^30^ reported that the bound fraction has chlorogenic acid content at a similar level to the free fraction. During *in vitro* digestion conditions, the degradation and polymerization of phenolics and their release from the matrix would occur simultaneously. For example, from the gastric to intestinal stage, the ferulic acid and ellagic acid contents of fresh and FD prunes were increased, while their content in the other samples was decreased (Table 4). The content of gallic acid increased in all samples except FD prunes, whose concentration did not change between the digestion steps.

The bioaccessibility of neochlorogenic, chlorogenic, and caffeic acids in fresh plums were 0.65, 3.74, and 20.29%, respectively. While Yu et al.^29^ determined the bioaccesibility of chlorogenic acid in plums as 23.11%, Seke et al.^30^ could not determine the chlorogenic acid in the digested plums. The bioaccessibility of caffeic acid in plums was reported as 19.10% by Seke et al.^30^ Considering both the amount of phenolics reached in the small intestinal phase and the bioaccessibility index (%) together, FD provided higher values compared to those of fresh plum and other dried samples. Among dried samples, considering the amount of those phenolics at the intestinal stage, the highest concentration was detected in FD prunes, followed by HAD samples. The bioaccessibility of neochlorogenic, chlorogenic, and caffeic acids in FD prunes was 5.59, 45.02, and 43.69% and 2.49, 22.20, and 16.18% in HAD prunes, respectively (Table 4). Our results confirmed that the structural changes that occurred during the drying affected the release of phenolics from the food matrix. Although the highest bioaccessibility was encountered for US-VD prunes (such as 54.89% for neochlorogenic acid, 45.58% for chlorogenic acid, and 73.05% for caffeic acid), it was due to their comparably lower initial values in the nondigested samples, not because of the amount available in the intestinal stage.

Similar to our findings, it was 38.48% for fresh grapes and increased to 110.18% by vacuum-drying.^25^ The level of individual phenolics in the residues after digestion is given in Table 3. Our results showed that all samples yielded a very low content of individual phenolics in the free phenolic fraction of the digested residue. Except for gallic and chlorogenic acids, none of the individual phenolics were detected in the digested residue of fresh plum. The ratio of the individual phenolics measured in the residue to those of the undigested sample ranged from 1.43 to 8.49% in the dried samples except US-VD prunes (14.89–31.03%) because of the lower initial level of phenolics in the undigested sample. Although *in vitro* digestion models were useful tools for assessing the proportion of food constituents that were released from the matrix, which then became available for absorption, they did not entirely replicate the complex physicochemical and physiological processes that occur in the human digestive tract.^77^

Bobrich et al.^27^ proposed that a meal of two large plums (∼ 200 g) would easily provide up to 140 mg of bound phenolics, and these plant matrix/cell wall-bound phenolics are transported to the colon without further degradation or absorption. The polyphenols that cannot be extracted from the matrix in the stomach and small intestine travel through the gastrointestinal tract as insoluble substrates, reaching the colon, where they release single polyphenols and different bioavailable metabolites through the action of bacterial microbiota.^78^

**Figure 2:**
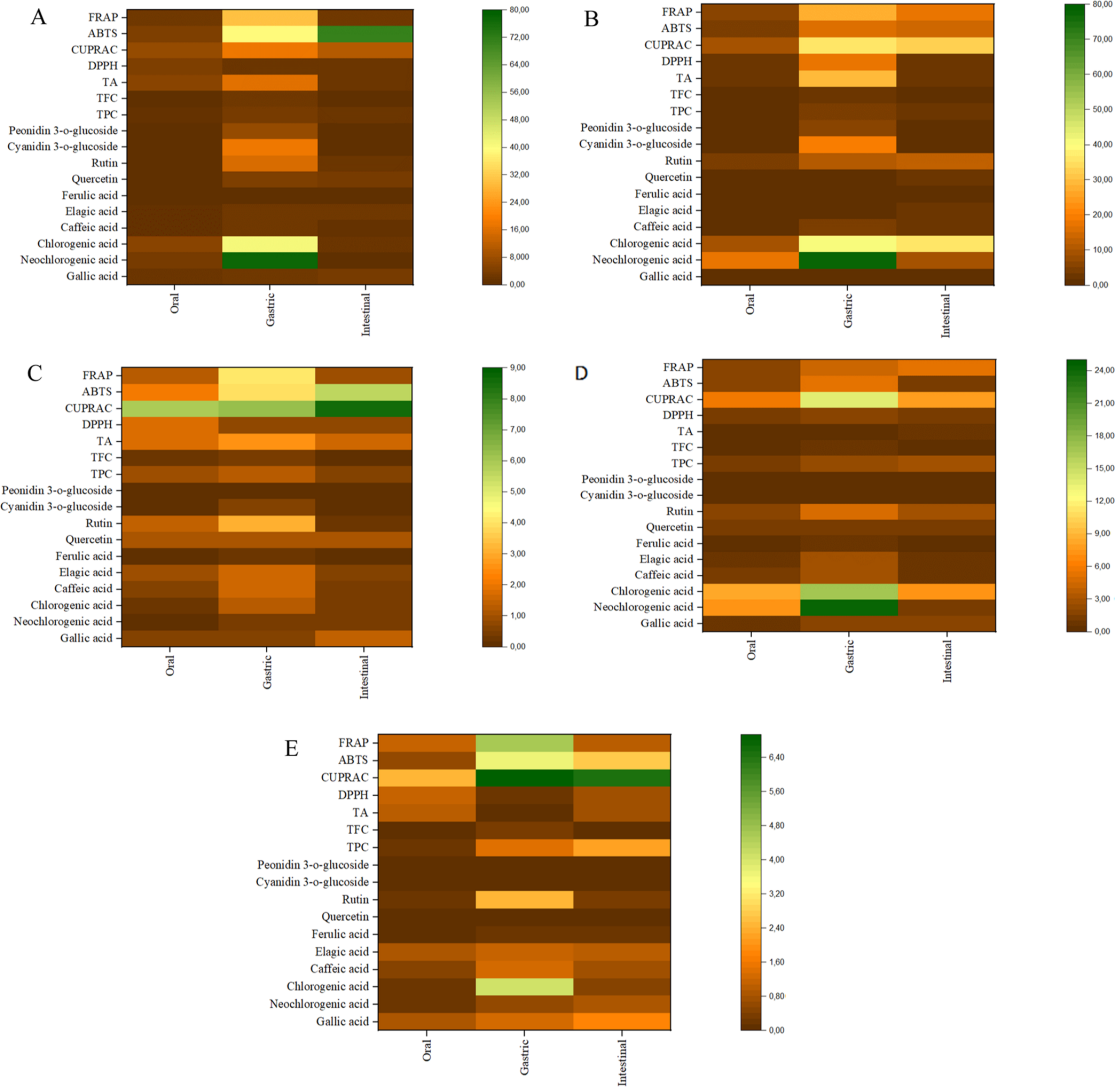
Heat maps representing the change of concentrations of individual phenolic compounds, TPC, TFC, TA, DPPH, CUPRAC, ABTS, and FRAP in the oral, gastric, and intestinal phase of fresh [**A**], FD [**B**], VD [**C**], HAD [**D**], and US-VD [**E**] samples. HAD, hot-air-drying; VD, vacuum-drying; US-VD, ultrasound-assisted vacuum-drying; and FD, freeze-drying. TPC; total phenolic content, TFC; total flavonoid content, TA; total monomeric anthocyanins, DPPH: 2,2-diphenyl-1-picrylhydrazyl radical scavenging activity, CUPRAC: copper reducing antioxidant capacity, ABTS: 2,2′-azino-bis (3-ethylbenzothiazoline6-sulfonic acid), and FRAP ferric reducing antioxidant power.

Additionally, a heat map was created (Figure 2) to provide a comprehensive visual representation of the change in concentrations of individual phenolics, TPC, TFC, TA, and antioxidant activities during each phase of *in vitro* digestion (oral, gastric, and intestinal) for both fresh and dried plums. Colors in the heat maps exhibited the intensity of the amount of phenolic compounds and antioxidant activity during various stages of *in vitro* digestion. The colors ranged from brown for low levels of phenolic compounds and antioxidant activity to green for high levels of phenolic compounds and antioxidant activity. After oral digestion, the concentration of neochlorogenic acid, which was represented by the green color, was the highest in fresh, HAD, and FD samples. On the other hand, the antioxidant activity (ABTS assay) of fresh and VD samples was highest in the intestinal stage, as shown by the green color.

## 4 Conclusions

In terms of the color, rehydration capacity, and retention of initial phenolic compounds, the most favorable drying method was freeze-drying (FD), followed by vacuum-drying (VD) and hot-air-drying (HAD). Ultrasound-assisted vacuum-drying (US-VD) was found to be the least desirable method for the dehydration of plums. The morphology and structure of the prunes differed depending on the drying method, which could potentially influence how bioactive components were released into the simulated digestion model. The changes in phenolic compounds in plums varied throughout the digestion stages, depending on the state of the food (fresh or dried) and the drying method employed. Based on the bioaccessibility index of measured properties (phenolics and antioxidant activities) in digested samples, FD prunes yielded the highest values, followed by HAD samples. In this study, only HPLC-DAD could have been employed to measure the changes of individual phenolics during digestion. Due to the complexity of phenolic compounds and their possible transformation products during gastrointestinal digestion, more comprehensive determination methods such as LC-MS/MS could be utilized for further research to identify the unknown constituents by providing information about the structural and molecular weight characterization. Further research should also examine the changes in free and bound phenolics in plum residues that remain after each digestion step, as well as the effects of predrying treatment methods for removing the waxy components of plums on the status of bioactive components during *in vitro* digestion.

## Supporting Information Available

The proximate composition of fresh plums (Table S1); the change in color parameters of fresh and dried plums (Table S2); and rehydration rates of dried plums at 25 °C (A) and 50 °C (B): FD, freeze-drying; VD, vacuum-drying; HAD, hot-air-drying; US-VD, ultrasound-assisted vacuum-drying (Figure S1) (PDF)
