# Supplementary material for: The Effects of Different Drying Methods on the In Vitro Bioaccessibility of Phenolics, Antioxidant Capacity, and Morphology of European Plums (Prunes domestica L.)
Source: ACS Omega. 2024 Mar 8;9(11):12711–24. doi: 10.1021/acsomega.3c08383 (PMC10955707; doi:10.1021/acsomega.3c08383)
Supplement: Supplementary file 1 — ao3c08383_si_001.pdf [file ao3c08383_si_001.pdf]

Supporting Information

The effects of different drying methods on the *in vitro* bioaccessibility of phenolics, antioxidant capacity, and morphology of European plums (*Prunes domestica* L*)*

Running title: *In vitro* bioaccessibility of fresh and dried plums

Elif Yener[1,2], Oznur Saroglu[1], Osman Sagdic[1], Ayse Karadag[1,*]

[1] Department of Food Engineering, Faculty of Chemical and Metallurgical Engineering, Yildiz Technical University, 34210, Istanbul, Turkey

[2]Food Institute, TUBITAK Marmara Research Center, Gebze, Turkey

Contains

Table S1. The proximate composition of fresh plum.

Table S2. The change in color parameters of fresh and dried plum.

Figure S1. Rehydration rates of dried plums at 25°C (A) and 50°C (B). FD- Freeze-drying, VD-Vacuum-drying, HAD- Hot air-drying, US-VD- Ultrasound assisted vacuum drying.

Table S1. The proximate composition of fresh plum

	g /100g fw
Moisture	80.02±0.20
Fat	-
Protein	0.90±0.04
Carbohydrate	16.90±0.21
Dietary Fiber	1.86±0.05
Ash	2.20±0.17
Minerals	*mg /100g fw*
K	227.34±2.96
P	20.18±1.85
Mg	10.50±0.09
Ca	6.90±0.07
Na	0.52±0.00
Fe	0.31±0.00
Zn	0.17±0.02
Mn	0.10±0.02
Cu	0.06±0.00

Table S2. The change in color parameters of fresh and dried plum

	Color parameters			
	**L**	**a***	**b***	**ΔE**
Outer surface				
Fresh	$31.16±0.76^{b}$	$3.78±2.18^{a}$	$-3.70±1.45^{d}$	-
HAD	$30.23±0.68^{b}$	$1.34±0.40^{c}$	$-2.70±0.50^{cd}$	$2.67±0.94^{c}$
VD	$30.30±0.94^{b}$	$1.90±0.80^{bc}$	$-2.05±0.72^{bc}$	$2.64±0.56^{c}$
US-VD	$30.39±0.67^{b}$	$0.68±0.29^{c}$	$-0.79±0.38^{a}$	$4.34±0.82^{a}$
FD	$33.87±0.95^{a}$	$3.08±0.71^{ab}$	$-1.33±0.28^{ab}$	$3.79±0.46^{b}$
Inner surface				
Fresh	$41.79±3.34^{b}$	$-1.06±1.02^{c}$	$16.17±3.84^{b}$	-
HAD	$33.34±2.19^{c}$	$4.92±0.58^{a}$	$6.59±1.03^{c}$	$14.20±1.70^{b}$
VD	$39.94±3.87^{b}$	$5.22±2.03^{a}$	$13.20±6.29^{b}$	$7.86±1.11^{c}$
US-VD	$34.30±1.26^{c}$	$1.54±0.54^{b}$	$1.97±1.57^{c}$	$16.29±1.81^{b}$
FD	$61.52±1.20^{a}$	$1.89±1.50^{b}$	$27.27±2.20^{a}$	$22.93±1.92^{a}$

Data are expressed as mean±S.D. of triplicate measurements. Means with different letters in the same column are significantly different ($p< 0.05$). HAD: Hot air-drying VD: Vacuum-drying, US-VD: Ultrasound assisted vacuum drying, FD: Freeze-drying

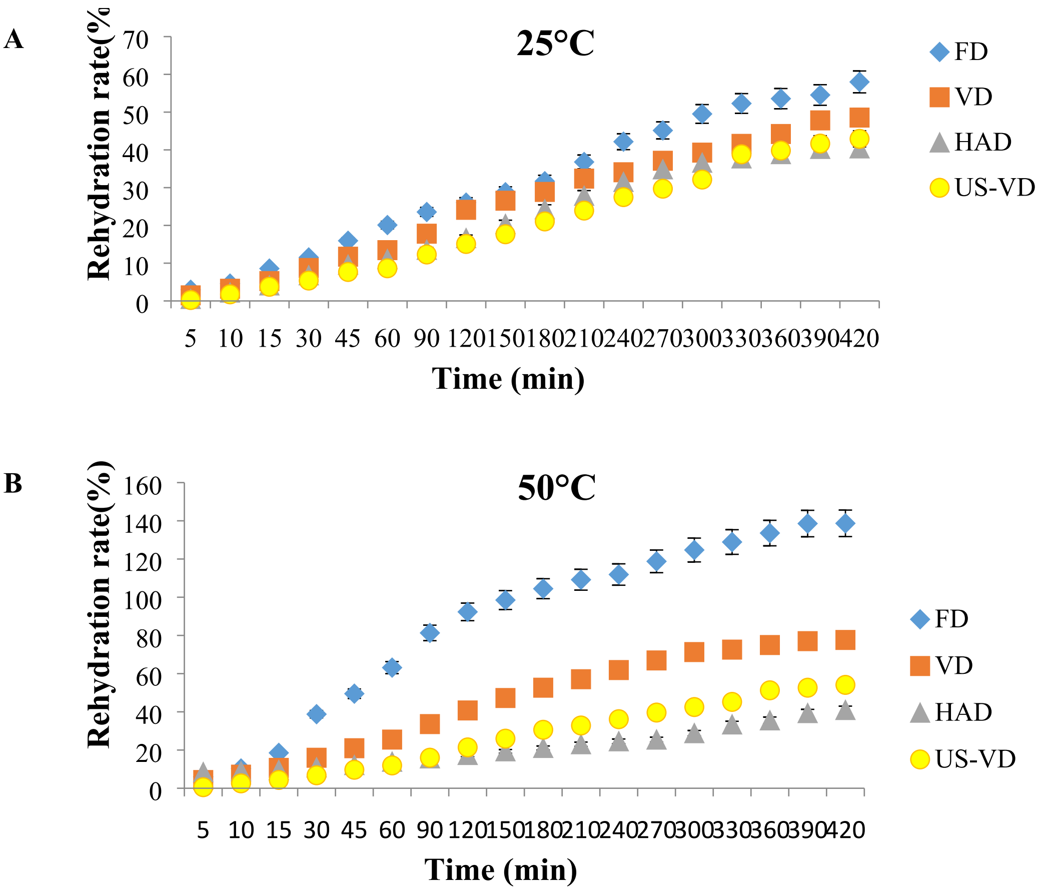

Figure S1 Rehydration rates of dried plums at 25°C (A) and 50°C (B). FD- Freeze-drying, VD-Vacuum-drying, HAD- Hot air-drying, US-VD- Ultrasound assisted vacuum drying.
